# Experience of racism in young people and future mental health in England: longitudinal analysis from the Next Steps Study

**DOI:** 10.1136/bmjment-2025-301668

**Published:** 2025-10-10

**Authors:** Hatem Mona, Rebecca Lacey, Ann John

**Affiliations:** 1 Swansea University - Singleton Park Campus, Swansea, UK; 2 City St George’s University of London, London, UK; 3 Swansea University Medical School, Swansea University, Swansea, West Glamorgan, UK

**Keywords:** Suicide & self-harm, Depression, Child & adolescent psychiatry, Depression & mood disorders, Data Interpretation, Statistical

## Abstract

**Background:**

Experience of racism is considered a predictor of ill health and poor well-being at all ages. Few studies examining the relationship between racism, mental health and self-harm are longitudinal. The aim of this study was to examine these associations longitudinally among youth in England.

**Methods:**

The data were obtained from the Next Steps Longitudinal Study on participants born in 1989–1990 in England. Waves 4 (2007) and 8 (2015) were used to measure associations between experiencing racism at age 17 and mental health outcomes at age 25. Logistic regression analyses were conducted. Multiple imputation was used to address missing data.

**Findings:**

Compared with those who did not experience racism, participants who experienced racism at 17 years scored 0.58 (95% CI 0.16 to 1.00) points higher in psychological distress (General Health Questionnaire-12) at age 25. No clear associations were found for overall life satisfaction (OR 1.06, 95% CI 0.85 to 1.34, p=0.597), self-harm (OR 0.79, 95% CI 0.40 to 1.56, p=0.494) or longstanding mental illness (OR 1.31, 95% CI 0.80 to 2.13, p=0.280).

**Conclusion:**

Exposure to racism at youth increased the risk of psychological distress, but not life satisfaction, self-harm or longstanding mental illnesses for young adults in England. Ongoing and future longitudinal studies exploring racism and mental health should incorporate electronic health records and validated measures of racism to better understand its effects on mental health across the life course.

**WHAT IS ALREADY KNOWN ON THIS TOPIC**

- Evidence of potentially negative impacts of experiences of racism in youth on the well-being, mental health and self-harm behaviours of young adults exists, but most research from the UK is cross-sectional.

**WHAT THIS STUDY ADDS**

- This study produces longitudinal evidence that exposure to racism at 17 years increased the risk of psychological distress at 25 years of age in England.

**HOW THIS STUDY MIGHT AFFECT RESEARCH, PRACTICE OR POLICY**

- A whole school approach to reduce racism at a school level including curriculum changes and training and awareness for teachers is required.
- Future and current cohort studies should be linked to health and administrative data to reduce missingness, although much methodological work is needed to reduce bias.

## Background

 Racism is defined as ‘the relegation of people of colour to inferior status and treatment based on unfounded beliefs about innate inferiority, as well as unjust treatment and oppression of people of colour whether intended or not’.^1^ While experiencing racism is increasingly recognised as being associated with poor well-being, mental health and self-harm, existing literature is mainly focused on adults.^2^ Adolescence is a critical developmental period characterised by sensitivity to social interactions, and therefore, exposure to racism at this stage may have long-term impacts later in life.^3^ Yet, experiencing racism in school remains the predominant form of racism affecting young people. It is considered a chronic stressor on the mental health of ethnic minorities since it may result in the anticipation and presumption of verbal and physical racism arising from previous events of trauma, being ignored or disadvantaged.^4^ In 2018, one US report found that, in all states, 63% of all school-related hate and bias incidents were racial or ethnic.^5^ In fact, students of racial and ethnic minorities have a higher likelihood of reporting negative school experiences and have worse perceptions of the quality of their schools.^6^

The literature points to a consistent relationship between racial discrimination and adverse mental health outcomes such as depression and/or anxiety.^7^ Discrimination is also associated with increased engagement in health-harming behaviours (like smoking and drug use), which are often employed as coping mechanisms in response to stress. These behaviours, in turn, are associated with poorer mental health, raising the possibility that they may lie on the causal pathway.^8^ Furthermore, having multiple time points of exposure to racism is associated with worse overall mental health, suggesting an accumulated effect.^8^ Some studies found sex or ethnicity to modify this relationship,^9^ but most explored US samples.

Many studies on racism and health are not considered ethnically diverse or representative of minority groups, and the tools used to measure racism can sometimes be inadequate or unvalidated in youth.^10^ Literature on racism and health is predominantly cross-sectional with a few longitudinal studies, most of which were conducted in the USA. These cross-sectional studies primarily have small sample sizes and few test for effect modification. Finally, among the longitudinal studies, barely any follow the mental health of adolescents into early adulthood. Therefore, little is known about the longitudinal effect of discrimination on mental health of young people.

To address these gaps, this study examined the association between racism among youth (at age 17) and mental health and self-harm (at age 25) in England using the Next Steps longitudinal cohort and tested for modification by sex and ethnicity.

## Method

### Data source

Next Steps is a national longitudinal cohort study following young people born between 1989 and 1990 in England.^11^ It provides data on secondary school children aged 14 and their transition into adulthood, with special focus on deprived schools. At wave 1, the study recruited over 15 000 participants aged 13–14 from both state-funded and fee-paying schools using a two-stage probability proportional to size sampling procedure with school serving as the primary sampling unit.^11^ Ethnic minority groups were oversampled to provide an adequate sample size for analysis. In wave 4, an ethnic minority boost was implemented, adding 352 Black Caribbean and Black African pupils.^11^

### Measures

Racism was our binary exposure, measured at wave 4 (2007, age 17) using the question ‘whether the young person has been threatened or insulted in the last 12 months due to skin colour or ethnicity’. In addition, four different mental health outcomes were taken from wave 8 (2015, age 25); General Health Questionnaire-12 (GHQ-12) score, overall life satisfaction, self-harm and the presence of a longstanding mental illness.

GHQ-12 is a non-intrusive validated screening tool for identifying individuals with mild psychological distress who may be at risk of developing psychiatric disorders with higher scores indicating more psychological distress.^12^ Psychological distress is defined as ‘a state of emotional suffering characterized by symptoms of depression (e.g., lost interest; sadness;) and anxiety (e.g., inability to relax, feeling tense)’.^13^ The range of possible values for the continuous GHQ-12 outcome is 0–12 given the standard coding method (0-0-1-1) used in the Next Steps study. Overall Life satisfaction is a self-reported measure and was categorised on a Likert scale from 1 to 5, with 1 being ‘very satisfied’ to 5 being ‘very dissatisfied’.

A binary self-harm outcome was derived from the question ‘whether participant has self-hurt on purpose in the past year?’. Finally, longstanding mental illness (binary) was derived from two questions in the questionnaire: ‘Whether the participant has a longstanding illness?’ and a follow-up question of ‘whether the effect of longstanding illness is due to mental health?’. This measure can be understood as the presence of a diagnosed and chronic mental health illness in comparison with a validated screening tool like GHQ-12.

### Covariates

We adjusted for a number of potential demographic and health-harming behaviour covariates including ethnicity, sex, religion, employment, child’s education/work status, parental level of education and total parental gross income at wave 4, and alcohol use, smoking and body mass index (BMI) at wave 8. Also, pre-exposure GHQ-12 from wave 2 (age 15) was adjusted to control for mental health status.

Ethnicity was recoded into five categories (1: white, 2: mixed, 3: Asian, 4: Black African or Black Caribbean, 5: other ethnic groups). Sex was coded as (1: male, 2: female). Seven religion categories were merged into four categories because of low counts in some categories. Religion was coded as (1: none, 2: Christian, 3: Muslim, 4: other). Also, the participants’ education/work status at age 17 was measured and coded as (1: doing A levels or similar course at school or college, 2: doing an apprenticeship or other type of training course, 3: in a full-time job, 4: something else).

Education was derived from the variable ‘The highest qualification held in family by either main or second parent’ categorised as having (1: higher education or degree level or equivalent; 2: general certificate of education (GCE) A level or equivalent; 3: general certificate of secondary education (GCSE) grades A-C or equivalent; 4: qualification at level 1 or below; and 5: no qualifications). It is important to note that GCSEs were introduced in 1988, therefore, many parents in this cohort would have completed earlier qualifications such as O levels or certificate of secondary education (CSEs). The categorisation aligns these qualifications with current frameworks for comparability. Specifically, Level 1 qualifications are equivalent to GCSE grades G-D or 3–1 in the current grading system.^14^ Also, the total gross income for both parents was banded in 12 categories. Due to some low counts, categories were merged (1: up to £10 399; 2: £10 400 to £20 799; 3: £20 800 to £36 399; 4: £36 400 to £51 999; and 5: £52 000 or more). Similarly, employment was recoded as a binary variable from the question ‘Whether you are currently employed?’ (1: yes; 2: no).

Two health behaviour covariates were measured: alcohol use and smoking. Participants were asked ‘How often do you have a drink containing alcohol?’ and ‘whether participant ever smoked cigarettes and how regularly?’ with a scale for drinking alcohol as (1: never; 2: monthly or less; 3: 2–4 times a month; 4: 2–3 times a week; 5: 4 or more times a week) and smoking as (1: never smoked; 2: used to smoke; 3: smokes occasionally; 4: smokes cigarettes every day). Finally, BMI was kept as a continuous variable and included in the models.

### Statistical analysis

To test the longitudinal association between reporting racial discrimination and the outcomes, linear (for GHQ-12), ordinal (for life satisfaction) and binary (for self-harm and longstanding mental illness) logistic regression were conducted. Missing data were imputed using multiple imputation by chained reaction under the assumption that data was missing at random and wave 8 survey weights were applied. For all outcomes, three models are tested. Model A showed the crude association, model B included all covariates except sex and ethnicity, and model C tested sex and ethnicity as effect modifiers in covariate-adjusted models. If sex/ethnicity did not improve the model as effect modifiers but as confounders, they were adjusted for in model C. To check whether interaction terms improved model fit, models with and without interaction terms were compared using Wald test via ‘mi test’ following multiple imputation estimation. This approach is appropriate for multiply imputed data and was applied across linear, binary and ordinal models. Full model outputs are provided in online supplemental tables 1-4. As a sensitivity check, complete case analysis was also performed (online supplemental table 5), including only participants with non-missing data on the exposure, all covariates and at least one outcome. All analyses were conducted using STATA V.18.^15^

## Results

### Descriptive results for the study sample

**Figure 1:**
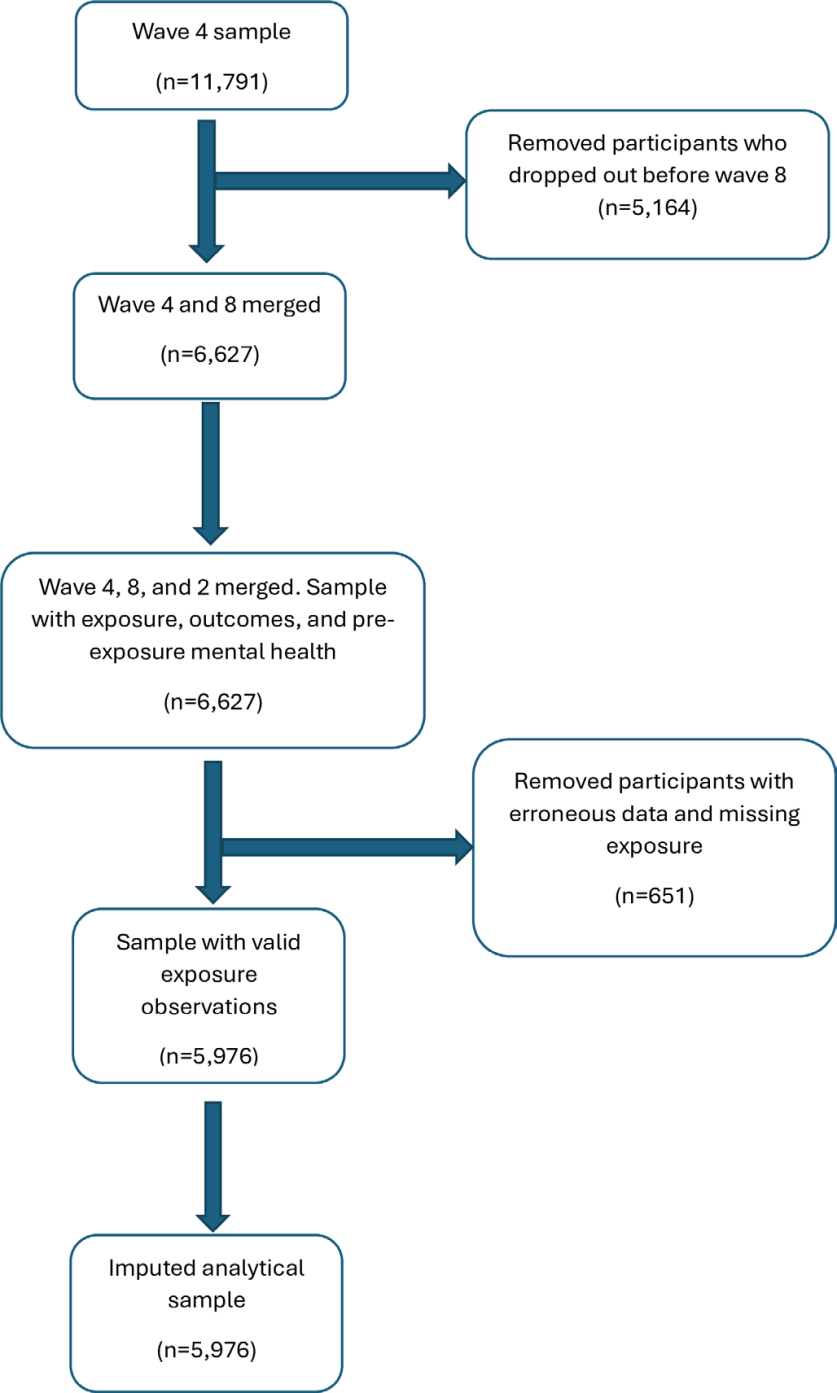
Flow chart of inclusion and exclusion.

Figure 1 shows the inclusion criteria used to arrive at the final analytical sample. After merging waves 2, 4 and 8 and applying exclusion (including removal of cases with missing exposure data), the analytical sample comprised 5976. Table 1 presents the descriptive statistics comparing the observed sample, with data on either the exposure or any of the four outcomes, and the analytical sample after imputation by chained reaction.

**Table 1:** Characteristics of the imputed analytical sample (N=5976) and the observed sample (n=6627)

		Observed sample	Analytical sample*
**Variables**	**Category**	**n (%)**	**N (%)**
***Exposure***			
**Threatened in the last 12 months due to skin colour?**	No Yes Missing	5894 (88.94) 563 (8.50) 170 (2.57)	5454 (91.27) 522 (8.73) –
***Outcomes***			
**Overall life satisfaction**	Very satisfied Fairly satisfied Neither nor Fairly unsatisfied Very unsatisfied Missing	1481 (22.35) 3256 (49.13) 1077 (16.25) 465 (7.02) 135 (2.04) 213 (3.21)	1379 (23.08) 3066 (51.31) 983 (16.45) 431 (7.21) 117 (1.96) –
**Whether participant has self-harmed in the past year?**	No Yes Missing	6085 (91.82) 218 (3.29) 324 (4.89)	5763 (96.44) 213 (3.56) –
**Longstanding mental illness**	No Yes Missing	5952 (89.81) 521 (7.86) 154 (2.32)	5510 (92.20) 466 (7.80) –
**GHQ-12**	Mean (range) SD	2.27 (0–12) 3.08	2.27 (0–12) 3.07
***Covariates***			
**Highest qualification of main or second parent**	Higher education or higher general certificate of education (GCE) A level general certificate of secondary education (GCSE) grades A-C Qualification at level one and below No qualification Missing	2416 (36.46) 1106 (16.69) 1529 (23.07) 460 (6.94) 982 (14.82) 134 (2.2)	2259 (37.80) 1046 (17.50) 1410 (23.59) 405 (6.78) 856 (14.32) –
**Total gross income of both parents**	Up to £10 399 £10 400 to £20 799 £20 800 to £36 399 £36 400 to £51 999 £52 000 or more Missing	699 (10.55) 1209 (18.24) 1460 (22.03) 1047 (15.80) 1036 (15.63) 1176 (17.75)	789 (13.20) 1391 (23.28) 1589 (26.59) 1106 (18.51) 1101 (18.42) –
**How often does the participant have an alcoholic drink?**	Never Monthly or less 2–4 times a month 2–3 times a week 4 or more times a week Missing	1417 (21.38) 1736 (26.20) 1912 (28.85) 1125 (16.98) 226 (3.41) 211 (3.18)	1264 (21.15) 1612 (26.97) 1809 (30.27) 1078 (18.04) 213 (3.56) –
**Whether ever smoked cigarettes and how regularly?**	Never smoked Used to smoke Smokes occasionally (not every day) Smokes every day Missing	3765 (56.81) 1029 (15.53) 716 (10.80) 899 (13.57) 218 (3.29)	3498 (58.53) 970 (16.23) 676 (11.31) 832 (13.92) –
**Religion**	Christian Muslim Other None Missing	3259 (49.18) 897 (13.54) 451 (6.81) 1950 (29.43) 70 (1.06)	3013 (50.42) 760 (12.72) 398 (6.66) 1805 (30.20) –
**Ethnicity**	White Mixed Asian Black African or Black Caribbean Other ethnic groups Missing	4611 (69.58) 306 (4.62) 1102 (16.63) 431 (6.50) 168 (2.54) 9 (0.14)	4251 (71.13) 269 (4.50) 949 (15.88) 362 (6.06) 145 (2.43) –
**Employment at age 25**	Employed Not employed Missing	5159 (77.85) 1453 (21.93) 15 (0.23)	4732 (79.18) 1244 (20.82) –
**Sex**	Male Female Missing	2938 (44.33) 3632 (54.81) 57 (0.86)	2714 (45.41) 3262 (54.59) –
**Education/work status at age 17**	Doing A levels or similar Doing apprenticeship or similar training In a full-time paid job Something else Missing	4908 (74.06) 625 (9.43) 293 (4.42) 640 (9.66) 161 (2.43)	4565 (76.39) 552 (9.24) 281 (4.70) 578 (9.67) –
**BMI (continuous**)	Mean (range) SD	25.24 (4.49–91.13) 5.70	25.22 (12.06–67.15) 5.41
**GHQ-12 (pre-exposure**)	Mean (range) SD	1.77 (0–12) 2.55	1.78 (0–12) 2.54

* Analytical sample: Participations with complete data on exposure, outcomes and all covariates (N=5976).

BMI, body mass index; GHQ-12, General Health Questionnaire-12.

In the study sample, (8.73%) of the participants were threatened due to skin colour in the last 12 months. In terms of outcomes, only (3.56%) and (7.80%) of young adults reported having self-harmed on purpose in the last year and having longstanding mental illness, respectively. When asked about their overall life satisfaction, the majority reported satisfaction, with 23.08% choosing very satisfied and 51.31% fairly satisfied. Only (7.21%) and (1.96%) chose fairly unsatisfied or very unsatisfied, respectively, and (16.45%) felt neither satisfied nor unsatisfied. The mean (SD) GHQ-12 score in the sample was 2.27 (3.07).

In all models, Wald test comparing interaction and non-interaction models showed no statistically significant improvement in fit (p>0.05). Consequently, sex and ethnicity were retained as covariates in the final models without interaction terms. All models were weighted using wave 8 survey weights and adjusted for pre-exposure mental health (measured using GHQ-12 at wave 2), in addition to other covariates.

### Psychological distress (GHQ-12)

Using linear regression, participants who were threatened due to skin colour scored 0.88 (95% CI 0.46 to 1.30, p<0.001) points higher than those who did not on the GHQ-12 (model A, table 2). After adjusting for religion, highest qualification of either parent, total gross income of both parents, smoking, alcohol use, employment at age 25, education/work status at age 17, BMI and pre-exposure GHQ-12 (model B, table 2), the coefficient slightly reduced to 0.55 (95% CI 0.14 to 0.97, p=0.009). After inclusion of sex and ethnicity as covariates, the association between racism and psychological distress became B=0.58 (95% CI 0.16 to 1.00, p=0.007).

**Table 2:** Weighted crude and adjusted models of the association between racism and mental health outcomes

Variable	Category	Model A*		Model B†		Model C‡	
**GHQ-12**							
		B	P value	B	P value	B	P value
**Racism**	No Yes	– 0.88 (0.46 to 1.30)	– <0.0001	– 0.55 (0.14 to 0.97)	– 0.009	– 0.58 (0.16 to 1.00)	– 0.007
**Overall life satisfaction**							
		OR	P value	OR	P value	OR	P value
**Racism**	No Yes	– 1.37 (1.10 to 1.71)	– 0.005	– 1.16 (0.93 to 1.46)	– 0.192	– 1.06 (0.85 to 1.34)	– 0.597
**Self-harm**							
		OR	P value	OR	P value	OR	P value
**Racism**	No Yes	– 1.05 (0.59 to 1.89)	– 0.858	– 0.76 (0.40 to 1.45)	– 0.404	– 0.79 (0.40 to 1.56)	– 0.494
**Longstanding mental illness**							
		OR	P value	OR	P value	OR	P value
**Racism**	No Yes	– 1.28 (0.82 to 2.01)	– 0.276	– 1.09 (0.67 to 1.78)	– 0.734	– 1.31 (0.80 to 2.13)	– 0.280

* Crude model.

† Adjusted for all covariates except sex and ethnicity.

‡ Model B+adjusting sex and ethnicity.

GHQ-12, General Health Questionnaire-12.

### Overall life satisfaction

Overall life satisfaction has five categories with referent category being ‘very satisfied’. Therefore, ordinal logistic regression was carried out while testing for the proportional odds assumption using the Brant test. Young people reporting racism had between 1.10 and 1.71 (OR=1.37, 95% CI 1.10 to 1.71, p=0.005) times higher odds of poorer life satisfaction than those not reporting racism (table 2, model A). Controlling for demographic and health-harming behaviour variables reduced the OR estimate to 1.16 (95% CI 0.93 to 1.46, p=0.192) but the association became non-significant. Similarly, after further adjusting for sex and ethnicity, the estimated adjusted OR for racism was 1.06 (95% CI 0.85 to 1.34, p=0.597). Although the association was non-significant, the proportional odds assumption was met (Brant test: p>0.05).

### Self-harm

Young adults who reported experiences of racism had slightly higher odds of self-harm in the last 12 months compared with those who did not (OR 1.05, 95% CI 0.59 to 1.89, p=0.858); however, this association was not statistically significant. After adjusting for additional covariates (table 2, model B), the OR attenuated to 0.76 (95% CI 0.40 to 1.45, p=0.404), and further adjustment for sex and ethnicity (table 2, model C) resulted in a similar estimate (OR=0.79, 95% CI 0.40 to 1.56, p=0.494). Across all models, there was no evidence of statistically significant association between experiences of racism and recent self-harm.

### Longstanding mental illness

The odds of having a longstanding mental illness at age 25 were 1.28 times higher among young people reporting racism, although it did not reach statistical significance (95% CI 0.82 to 2.01, p=0.276). Adjusting for several covariates (model B) reduced the OR to 1.09 (95% CI 0.67 to 1.78, p=0.734), but the association remained non-significant. In the fully adjusted model (table 2, model C), young adults reporting racism showed a 31% higher likelihood of having longstanding mental illness compared with those not subjected to racism. However, this association remained non-significant (OR 1.31, 95% CI 0.80 to 2.13, p=0.280).

## Discussion

In this longitudinal study, we found that experiences of racial discrimination at age 17 were associated with increased psychological distress at age 25, even after adjusting for sex, ethnicity, religion, highest qualification of either parent, total gross income of both parents, smoking, alcohol use, employment at age 25, education/work status at age 17, BMI and pre-exposure GHQ-12. Notably, no association was found between racism and overall life satisfaction, self-harm or having a longstanding mental illness. While previous longitudinal studies focused on shorter intervals or specific populations, our study provides evidence of the enduring impact of racial discrimination on mental health outcomes by extending the observation period into adulthood and capturing the long-term effects of adolescent racial discrimination. In all cases, sex and ethnicity did not modify any of the tested associations. Instead, both acted as confounders except in the case of self-harm where only sex was a confounder.

Existing literature consistently demonstrates a significant association between experiences of racism and elevated levels of psychological distress.^16^,^18^ For instance, Gil-González *et al* found that men who experienced racism had 5.6 times higher odds (95% CI 3.9 to 8.2) of reporting poorer mental health (dichotomised GHQ-12 scores) compared with men who did not report such experiences.^16^ The association was also significant for women, though less pronounced, with an OR of 2.2 (95% CI 1.6 to 3.0), after controlling for educational level and employment status. However, this study focused solely on foreign-born adult participants and did not include immigrants born in Spain. Consequently, the observed associations may be stronger than those that would be found in a more comprehensive study that accounts for factors like access to resources, network and language proficiency.

Similarly, a cross-sectional study involving Aboriginal Australian adults (n=221) reported that individuals who experienced interpersonal racism had 2.66 times higher odds (95% CI 1.39 to 5.08, p=0.03) of experiencing high or very high psychological distress, as measured by the Kessler Psychological Distress scale, compared with those who did not report such experiences.^17^ It is important to note that the study’s sample size was relatively small, and participants were specifically questioned about racism in healthcare settings, which may limit the generalisability of the finding.

Moreover, Hackett *et al* found a longitudinal association between reporting discrimination by ethnicity or nationality and psychological distress measured in GHQ-12 2 years after baseline in adjusted models (B=0.52, 95% CI 0.20 to 0.85, p<0.01). The longitudinal design of this paper is a strong merit, although the effect size may be influenced by unmeasured confounding variables like social support, health behaviours, school-related and family-related measures.^18^

In contrast to prior research, our study did not find a statistically significant association between reported experiences of racism and life satisfaction at wave 8 after applying weights and adjusting other covariates. This finding diverges from several studies that have reported negative associations between experiencing racism and life satisfaction, as evidenced across different methodologies. For instance, Stronge *et al* used path analysis to show that, among adults, perceived discrimination was directly associated with reduced life satisfaction (B=−0.121, SE=0.019, p<0.001) and indirectly positively associated through ethnic identity (B=0.023, SE=0.005, p<0.001), suggesting that stronger ethnic identification may buffer the adverse effects of discrimination on life satisfaction.^19^

Another study employing cluster sampling found that adults experiencing racial discrimination had significantly higher odds of being dissatisfied or very dissatisfied with their lives compared with adults who did not report such experiences with an adjusted OR of 1.94 (95% CI 1.44 to 2.61) after controlling for sex, age, education and area deprivation.^20^ The study observed a dose-response relationship; individuals who experienced one additional form of discrimination had an adjusted OR of 2.04 (95% CI 1.24 to 3.32) and those with two or more additional forms had an adjusted OR of 2.74 (95% CI 1.89 to 3.96) indicating that multiple forms of discrimination compound a negative impact on life satisfaction. However, our findings align more closely with those of Barnes *et al*, who found no significant association between perceived discrimination and life satisfaction among African American college students.^21^ As noted by the authors, specific demographic contexts of college students or the use of multi-item measures may account for lack of observed association. In our study, the lack of association may reflect adjustment for pre-exposure mental health, nuances of applying weighting or potentially unmeasured factors such as community belonging or coping styles which were not measured in the Next Steps Study.

Although our study did not find a significant association between reported racism and self-harm in fully adjusted weighted models, this area remains underexplored in the literature. Some prior studies have suggested a possible link. For example, a study on immigrant adolescents in Sweden found an association between being exposed to harassment due to ethnicity and self-harming behaviours including intentionally cutting and burning parts of the body (β=0.07, 95% CI 0.03 to 0.13, p=0.01) controlling for sex.^22^ While race and ethnicity are often overlapping concepts, they are not interchangeable. Individuals of the same race can belong to different ethnic groups and vice versa. Another study found that discrimination-related stress was associated with increased self-harming behaviours (p<0.01) and suicidal ideation (p<0.05) among males, but not females,^23^ after adjusting for 7 other types of stressors. Similarly, Jamieson *et al* observed that Aboriginal Australian young adults who reported unfair treatment had a higher likelihood of having suicide ideation (B=0.34, 95% CI 0.08, 0.60) compared with those who did not.^24^ Our findings do not align with these results, which may be due to differences in population characteristics, study design, cultural context or the inclusion of important confounders such as baseline mental health.

Experiences of racism are rarely studied in relation to longstanding mental illnesses. However, some literature exists on its association with chronic physical or combined health illnesses. Hackett *et al* adjusted for sex, income, education and ethnicity and found that reporting discrimination was associated with 31% (OR = 1.31, 95% CI 1.01 to 1.69, p<0.05) higher likelihood of having a limiting longstanding illness compared with not reporting discrimination (18). One study found that only among Latino-Americans was there an association between perceiving discrimination and chronic pain conditions (OR 1.69; 95% CI 1.14 to 2.52).^25^ One reason our study did not yield an association could be due to the small cell numbers for participants who reported having a longstanding metal illness. Another issue is that, in most cases, studies either measure all forms of discrimination together and do not focus on racism per se, or the health outcome is measured using questionnaires of psychological well-being.

While some studies have identified sex and ethnicity as potential moderators of the association between racism and mental health outcomes,^4 5 9^ our analysis did not find significant moderation effects of these variables. This can be attributed to several factors. For example, small sample sizes within certain subgroups may have been insufficient to detect interaction effects, hence limiting statistical power. Also, ethnicity was categorised in a way that used broad categories which may not capture the experiences of individuals of intersecting identities. The intersection of sex and ethnicity suggests that individuals may experience compounded forms of discrimination that could have been tested with joint stratification. However, small sample sizes of subgroups were the main reason preventing that. One paper examined ethnic identity as a moderator between discrimination and well-being and found that it was neither reinforcing nor protecting the youth in the study from the effects of discrimination.^26^ Other research found a protective effect of ethnic identification between racial discrimination and self-esteem, with youth reporting a strong connection to their ethnic group having higher self-esteem^27^ and reduced behaviour problems^28^ than their counterparts.

### Strengths and limitations

Due to the lack of literature on racism and mental health among the young people in the UK, this paper established longitudinal evidence of this association. Most of the literature studied middle-aged, elderly or child participants and very few came from cohort studies. Assessing the effect of racism in youth on, later-in-life, mental health provides a more extensive view of potential school-related peer-to-peer and outside of school racism and its accumulated effects on mental health. Additionally, the Next Steps Longitudinal Study is representative of all school children in England of a similar age. Most other studies on the topic had small sample sizes focusing on the more negatively impacted ethnicities but not comparing different ethnicities.

A few limitations exist in this study. The variable ‘racism’ was dichotomised by design and without measuring its frequency. Measuring the frequency of racism would allow for analysing its accumulation. Also, the questionnaires do not mention whether the subjected discrimination happened at school, outside of it or both. Understanding the source of racism is essential for recommending social-level or school-level interventions.

A key limitation of this study was the presence of attrition and non-response bias, which may affect both internal and external validity. One paper pointed out that attrition and non-response biased the socioeconomic composition of the sample as individuals from lower socioeconomic backgrounds were more likely to drop.^29^ In our study, missing data may have been associated with sociodemographic characteristics and outcomes; however, following multiple imputation and application of survey weights, comparison between the imputed analytical sample and the observed sample with missingness revealed minimal differences. This supports the assumption that data were missing at random and suggests that imputation helped to reduce bias and preserve representativeness.

Additionally, our inclusion of a pre-exposure mental health measure (GHQ-12) strengthens our interpretation by adjusting for baseline mental health differences. Nevertheless, while imputation improves robustness, it does not fully eliminate the risk of residual confounding or measurement error, which may still lead to underestimation or overestimation of effects.

### Implications

To better understand the impact of racism across the life course, it is imperative that future and ongoing cohort studies incorporate detailed questionnaire items addressing both the exposure to and frequency of racist experiences. Such research may capture unobserved pathways and improve understanding of the long-term mental health impacts of racism. Furthermore, linkage to health and administrative data can enrich analyses by providing objective measures of well-being and mental health outcome, potentially mitigating issues related to self-reporting and missing data. However, it is important to acknowledge the limitation of data linkage since not all participants in longitudinal studies consent to data linkage, hence leading to reduced sample sizes and potential selection biases.

At the educational level, a culture of counter-racism needs to be implemented to tackle institutional racism. Some argue that school-related policies do not stress the impacts of racism enough.^30^ Policies like school uniform do not take into account the clothing of certain ethnic minorities and hence leave some students feeling outside of the norm. Additionally, school curriculum does not emphasise learning about the colonial period or its impacts on ethnic minorities in the western world. Therefore, the curriculum needs changes in syllabus, resources and teacher’s literacy.^30^

## Conclusion

In conclusion, being subjected to racism can have lasting mental health effects on young people as they enter early adulthood. Those exposed to racial discrimination reported higher psychological distress, while other aspects of mental health such as life satisfaction and self-harm did not show strong patterns in our findings. National and school-specific policies are needed to prevent racist occurrences targeting ethnic minorities. As this issue can persist over the life course, ongoing support and action are essential at different stages to protect mental health and well-being.

## Supplementary material

10.1136/bmjment-2025-301668 online supplemental file 1

## Data Availability

Data may be obtained from a third party and are not publicly available.
