# Supplementary material for: Experience of racism in young people and future mental health in England: longitudinal analysis from the Next Steps Study
Source: BMJ Ment Health. 2025 Oct 10;28(1):e301668. doi: 10.1136/bmjment-2025-301668 (PMC12516973; doi:10.1136/bmjment-2025-301668)
Supplement: online supplemental file 1 [file bmjment-28-1-s001.docx]

## Supplementary material

**Supplementary table 1: Weighted adjusted models with and without interaction terms for of the association between racism and GHQ (coefficients table)**

| Variable | | With interaction terms | | Without interaction terms | |
| --- | --- | --- | --- | --- | --- |
|  |  | **Β (C.I)** | **p-value** | **Β (C.I)** | **p-value** |
| Racism | **Yes** | **0.71**  **(0.02, 1.40)** | **0.045** | **0.58**  **(0.156, 1.00)** | **0.007** |
| Sex | **Female** | **0.06**  **(-0.15, 0.28)** | **0.570** | **0.06**  **(-0.15, 0.26)** | **0.590** |
| Race x Sex | **Yes x Female** | **-0.04**  **(-0.86, 0.77)** | **0.915** | **-** | **-** |
| Ethnicity | **Mixed**  **Asian**  **Black African or Black Caribbean**  **Other ethnic group** | **-0.05**  **(-0.63, 0.53)**  **-0.33**  **(-1.00, 0.35)**  **-0.08**  **(-0.68, 0.52)**  **0.53**  **(-0.35, 1.41)** | **0.869**  **0.345**  **0.794**  **0.234** | **-0.059**  **(-0.65, 0.53)**  **-0.31**  **(-0.98, 0.37)**  **-0.15**  **(-0.68, 0.37)**  **0.32**  **(-0.48, 1.12)** | **0.842**  **0.375**  **0.572**  **0.433** |
| Race x Ethnicity | **Yes** **x Mixed**  **Yes x Asian**  **Yes x Black African or Black Caribbean**  **Yes x Other ethnic group** | **-0.14**  **(-1.83, 1.54)**  **0.06**  **(-0.89, 1.00)**  **-0.44**  **(-1.52, 0.64)**  **-1.60**  **(-3.03, -0.16)** | **0.869**  **0.902**  **0.425**  **0.029** | **-** | **-** |
| Religion | **Christian**  **Muslim**  **Other religion** | **0.13**  **(-0.09, 0.35)**  **-0.44**  **(-1.16, 0.29)**  **0.08**  **(-0.66, 0.83)** | **0.255**  **0.236**  **0.831** | **0.13**  **(-0.09, 0.35)**  **-0.44**  **(-1.16, 0.29)**  **0.10**  **(-0.64, 0.85)** | **0.242**  **0.240**  **0.787** |
| Income | **£10,400 to £20,799**  **£20,800 to £36,399**  **£36,400 to £51,999**  **£52,000 or more** | **0.15**  **(-0.27, 0.56)**  **0.21**  **(-.23, 0.64)**  **-0.12**  **(-0.56, 0.31)**  **-0.20**  **(-0.66, 0.26)** | **0.482**  **0.347**  **0.579**  **0.391** | **0.14**  **(-0.27, 0.56)**  **0.20**  **(-0.24, 0.64)**  **-0.13**  **(-0.57, 0.30)**  **-0.21**  **(-0.67, 0.25)** | **0.505**  **0.367**  **0.546**  **0.368** |
| Parental education | **GCE A level**  **GCSE grades A-C**  **Qualification at level 1 and below**  **No qualification** | **-.173**  **(-0.44, 0.10)**  **-.153**  **(-0.41, 0.11)**  **.182**  **(-0.28, 0.65)**  **-.09**  **(-0.54, 0.35)** | **0.208**  **0.248**  **0.441**  **0.685** | **-0.18**  **(-0.45, 0.09)**  **-0.15**  **(-0.41, 0.11)**  **0.19**  **(-0.28, 0.65)**  **-0.09**  **(-0.54, 0.35)** | **0.193**  **0.255**  **0.433**  **0.682** |
| Smoking | **Used to smoke**  **Smokes occasionally (not every day)**  **Smokes everyday** | **0.256**  **(0.02, 0.53)**  **.297**  **(-0.01, 0.60)**  **0.504**  **(0.20, 0.81)** | **0.067**  **0.055**  **0.001** | **0.26**  **(-0.01, 0.53)**  **0.29**  **(-0.02, 0.59)**  **0.51**  **(0.20, 0.81)** | **0.063**  **0.064**  **0.001** |
| Alcohol use | **Monthly or less**  **2-4 times a month**  **2-3 times a week**  **4 or more times a week** | **-0.41**  **(-0.80, -0.01)**  **-0.58**  **(-0.97, -0.19)**  **-0.82**  **(-1.23, -0.40)**  **-0.14**  **(-0.77, 0.48)** | **0.043**  **0.003**  **<0.001**  **0.658** | **-0.41**  **(-0.81, -0.02)**  **-0.59**  **(-0.98, -0.20)**  **-0.82**  **(-1.23,** **-0.40)**  **0.14**  **(-0.77, 0.48)** | **0.039**  **0.003**  **< 0.001**  **0.657** |
| Employment at age 25 | **No** | **0.72**  **(0.45, 0.99)** | **0.000** | **0.71**  **(0.45, 0.98)** | **<0.001** |
| Education/work status at age 17 | **Doing apprenticeship or similar training**  **In a full-time paid job**  **Something else** | **-0.16**  **(-0.49, 0.16)**  **-0.81**  **(-1.23, -0.39)**  **0.04**  **(-0.33, 0.41)** | **0.328**  **<0.001**  **0.825** | **-0.16**  **(-0.48, 0.17)**  **-0.81**  **(-1.23, -.39)**  **0.04**  **(-0.33, 0.42)** | **0.336**  **<0.001**  **0.820** |
| BMI | **-** | **.010**  **(-0.01, 0.03)** | **0.300** | **0.01**  **(-0.01, 0.03)** | **0.296** |
| GHQ-12 (pre-exposure) | **-** | **.252**  **(0.21, 0.30)** | **<0.001** | **0.25**  **(0.21, 0.30)** | **0.000** |

**Supplementary table 2: Weighted adjusted models with and without interaction terms for of the association between racism and overall life satisfaction (coefficients table)**

| Variable | | With interaction terms | | Without interaction terms | |
| --- | --- | --- | --- | --- | --- |
|  |  | **Β (C.I)** | **p-value** | **Β (C.I)** | **p-value** |
| Racism | **Yes** | **0.16**  **(-0.22, 0.54)** | **0.414** | **0.06**  **(-0.17, 0.29)** | **0.597** |
| Sex | **Female** | **-0.34**  **(-0.47, -0.21)** | **<0.001** | **-0.33**  **(-0.46, -0.20)** | **<0.001** |
| Race x Sex | **Yes x Female** | **0.09**  **(-0.35, 0.52)** | **0.693** | **-** | **-** |
| Ethnicity | **Mixed**  **Asian**  **Black African or Black Caribbean**  **Other ethnic group** | **0.19**  **(-0.16, 0.53)**  **0.45**  **(0.04, 0.87)**  **0.62**  **(0.30, 0.93)**  **0.78**  **(0.31, 1.24)** | **0.284**  **0.030**  **<0.001**  **0.001** | **0.10**  **(-.24, 0.44)**  **0.43**  **(0.03, 0.83)**  **0.57**  **(0.29, 0.85)**  **0.66**  **(0.24, 1.09)** | **0.564**  **0.037**  **<0.001**  **0.002** |
| Race x Ethnicity | **Yes** **x Mixed**  **Yes x Asian**  **Yes x Black African or Black Caribbean**  **Yes x Other ethnic group** | **-0.45**  **(-1.38, 0.47)**  **-0.16**  **(-0.67, 0.36)**  **-0.33**  **(-0.96, 0.30)**  **-0.84**  **(-1.73, 0.05)** | **0.336**  **0.555**  **0.303**  **0.064** | **-** | **-** |
| Religion | **Christian**  **Muslim**  **Other religion** | **-0.18**  **(-0.32, -0.05)**  **-0.52**  **(-0.92, -0.12)**  **-0.44**  **(-0.92, 0.03)** | **0.009**  **0.011**  **0.065** | **-0.17**  **(-0.31, -0.04)**  **-0.51**  **(-0.91, -0.11)**  **-0.42**  **(-0.89, 0.05)** | **0.011**  **0.012**  **0.079** |
| Income | **£10,400 to £20,799**  **£20,800 to £36,399**  **£36,400 to £51,999**  **£52,000 or more** | **-0.14**  **(-0.40, 0.11)**  **-0.17**  **(-0.44, 0.10)**  **-0.27**  **(-0.55, 0.01)**  **-0.52**  **(-0.82, -0.23)** | **0.272**  **0.212**  **0.064**  **0.001** | **-0.15**  **(-0.41, 0.10)**  **-0.17**  **(-0.44, 0.10)**  **-0.28**  **(-0.57, 0.01)**  **-0.53**  **(-0.83, -0.24)** | **0.247**  **0.213**  **0.057**  **<0.001** |
| Parental education | **GCE A level**  **GCSE grades A-C**  **Qualification at level 1 and below**  **No qualification** | **-0.08**  **(-0.25, 0.09)**  **-0.05**  **(-0.21, 0.11)**  **0.05**  **(-0.23, 0.33)**  **-0.05**  **(-0.31, 0.20)** | **0.359**  **0.540**  **0.739**  **0.688** | **-0.08**  **(-0.26, 0.09)**  **-0.05**  **(-0.21, 0.12)**  **0.05**  **(-0.23, 0.33)**  **-0.05**  **(-0.30, 0.25)** | **0.353**  **0.576**  **0.725**  **0.707** |
| Smoking | **Used to smoke**  **Smokes occasionally (not every day)**  **Smokes everyday** | **0.04**  **(-0.12, 0.20)**  **0.32**  **(0.13, 0.51)**  **0.52**  **(0.33, 0.71)** | **0.620**  **0.001**  **<0.001** | **0.04**  **(-0.12, 0.21)**  **0.32**  **(0.12, 0.51)**  **0.52**  **(0.33, 0.71)** | **0.604**  **0.001**  **<0.001** |
| Alcohol use | **Monthly or less**  **2-4 times a month**  **2-3 times a week**  **4 or more times a week** | **0.02**  **(-0.25, 0.25)**  **-0.06**  **(-0.29, 0.18)**  **-0.19**  **(-0.44, 0.06)**  **-0.06**  **(-0.29, 0.17)** | **0.894**  **0.640**  **0.137**  **0.728** | **0.01**  **(-0.22, 0.24)**  **-0.06**  **(-0.29, 0.17)**  **-0.19**  **(-0.44, 0.06)**  **-0.06**  **(-0.42, 0.29)** | **0.931**  **0.595**  **0.138**  **0.722** |
| Employment at age 25 | **No** | **0.41**  **(0.25, 0.56)** | **<0.001** | **0.40**  **(0.24, 0.56)** | **<0.001** |
| Education/work status at age 17 | **Doing apprenticeship or similar training**  **In a full-time paid job**  **Something else** | **-0.26**  **(-0.48, -0.05)**  **-0.14**  **(-0.41, 0.12)**  **0.08**  **(-0.15, 0.30)** | **0.015**  **0.297**  **0.479** | **-0.26**  **(-0.48, -0.05)**  **-0.14**  **(-0.40, 0.12)**  **0.08**  **(-0.14, 0.30)** | **0.016**  **0.292**  **0.481** |
| BMI | **-** | **0.01**  **(-0.00, 0.02)** | **0.251** | **0.01**  **(-0.00, 0.02)** | **0.252** |
| GHQ-12 (pre-exposure) | **-** | **0.09**  **(0.06, 0.11)** | **<0.001** | **0.09**  **(0.06, 0.11)** | **<0.001** |

**Supplementary table 3: Weighted adjusted models with and without interaction terms for of the association between racism and self-harm (coefficients table)**

| Variable | | With interaction terms | | Without interaction terms | |
| --- | --- | --- | --- | --- | --- |
|  |  | **Β (C.I)** | **p-value** | **Β (C.I)** | **p-value** |
| Racism | **Yes** | **-0.04**  **(-1.12, 1.04)** | **0.946** | **-0.24**  **(-0.92, 0.45)** | **0.494** |
| Sex | **Female** | **0.04**  **(-0.36, 0.43)** | **0.854** | **0.05**  **(-0.33, 0.44)** | **0.796** |
| Race x Sex | **Yes x Female** | **0.20**  **(-1.09, 1.50)** | **0.758** | **-** | **-** |
| Ethnicity | **Mixed**  **Asian**  **Black African or Black Caribbean**  **Other ethnic group** | **0.40**  **(-0.47, 1.27)**  **0.14**  **(-1.15, 1.43)**  **-0.81**  **(-1.86, 0.23)**  **0.97**  **(-0.27, 2.21)** | **0.370**  **0.831**  **0.128**  **0.123** | **0.05**  **(-0.84, 0.94)**  **0.12**  **(-1.11, 1.34)**  **-0.83**  **(-1.74, 0.09)**  **0.78**  **(-0.45, 2.01)** | **0.912**  **0.851**  **0.077**  **0.213** |
| Race x Ethnicity | **Yes** **x Mixed**  **Yes x Asian**  **Yes x Black African or Black Caribbean**  **Yes x Other ethnic group** | **-2.42**  **(-4.91, 0.07)**  **-0.21**  **(-1.60, 1.19)**  **0.25**  **(-2.26, 1.76)**  **-2.49**  **(-4.93, -0.06)** | **0.057**  **0.774**  **0.807**  **0.045** | **-** | **-** |
| Religion | **Christian**  **Muslim**  **Other religion** | **-0.20**  **(-0.57, 0.17)**  **-0.45**  **(-1.73, 0.84)**  **-1.28**  **(-2.57, 0.01)** | **0.297**  **0.496**  **0.052** | **-0.19**  **(-0.56, 0.18)**  **-0.43**  **(-1.71, 0.84)**  **-1.22**  **(-2.51, 0.08)** | **0.321**  **0.505**  **0.005** |
| Income | **£10,400 to £20,799**  **£20,800 to £36,399**  **£36,400 to £51,999**  **£52,000 or more** | **-0.05**  **(-0.72, 0.63)**  **0.18**  **(-0.54, 0.90)**  **-0.05**  **(-0.79, 0.68)**  **-0.14**  **(-1.00, 0.72)** | **0.896**  **0.618**  **0.883**  **0.752** | **-0.07**  **(-0.74, 0.60)**  **0.17**  **(-0.54, 0.89)**  **-0.07**  **(-0.80, 0.66)**  **-0.16**  **(-1.02, 0.70)** | **0.842**  **0.629**  **0.844**  **0.719** |
| Parental education | **GCE A level**  **GCSE grades A-C**  **Qualification at level 1 and below**  **No qualification** | **-0.30**  **(-0.82, 0.22)**  **-0.51**  **(-1.00, -0.01)**  **-0.07**  **(-0.76, 0.63)**  **-0.27**  **(-1.00, 0.47)** | **0.259**  **0.043**  **0.851**  **0.476** | **-0.31**  **(-0.83, 0.21)**  **-0.49**  **(-0.98, 0.00)**  **-0.05**  **(-0.74, 0.64)**  **-0.24**  **(-0.97, 0.49)** | **0.239**  **0.050**  **0.885**  **0.517** |
| Smoking | **Used to smoke**  **Smokes occasionally (not every day)**  **Smokes everyday** | **0.40**  **(-0.10, 0.90)**  **0.49**  **(-0.05, 1.02)**  **0.93**  **(0.47, 1.40)** | **0.117**  **0.074**  **<0.001** | **0.40**  **(-0.10, 0.90)**  **0.47**  **(-0.06, 1.01)**  **0.93**  **(0.47, 1.40)** | **0.115**  **0.083**  **<0.001** |
| Alcohol use | **Monthly or less**  **2-4 times a month**  **2-3 times a week**  **4 or more times a week** | **0.11**  **(-0.49, 0.71)**  **0.02**  **(-0.59, 0.63)**  **-0.17**  **(-0.82, 0.48)**  **0.36**  **(-0.47, 1.20)** | **0.727**  **0.944**  **0.610**  **0.395** | **0.12**  **(-0.48, 0.72)**  **0.02**  **(-0.59, 0.63)**  **-0.15**  **(-0.80, 0.50)**  **0.37**  **(-0.47, 1.21)** | **0.695**  **0.952**  **0.653**  **0.382** |
| Employment at age 25 | **No** | **0.84**  **(0.49, 1.19)** | **<0.001** | **0.82**  **(0.47, 1.17)** | **<0.001** |
| Education/work status at age 17 | **Doing apprenticeship or similar training**  **In a full-time paid job**  **Something else** | **-0.69**  **(-1.49, 0.11)**  **-1.42**  **(-2.64, -0.20)**  **0.33**  **(-0.16, 0.82)** | **0.090**  **0.023**  **0.183** | **-0.69**  **(-1.49, 0.10)**  **-1.41**  **(-2.64, -0.19)**  **0.32**  **(-0.17, 0.82)** | **0.088**  **0.024**  **0.195** |
| BMI | **-** | **0.03**  **(0.00, 0.06)** | **0.026** | **0.03**  **(0.00, 0.06)** | **0.025** |
| GHQ-12 (pre-exposure) | **-** | **0.19**  **(0.14, 0.24)** | **<0.001** | **0.19**  **(0.14, 0.24)** | **<0.001** |

**Supplementary table 4: Weighted adjusted models with and without interaction terms for of the association between racism and longstanding mental illness (coefficients table)**

| Variable | | With interaction terms | | Without interaction terms | |
| --- | --- | --- | --- | --- | --- |
|  |  | **Β (C.I)** | **p-value** | **Β (C.I)** | **p-value** |
| Racism | **Yes** | **0.58**  **(-0.13, 1.27)** | **0.109** | **0.27**  **(-0.22, 0.76)** | **0.280** |
| Sex | **Female** | **0.10**  **(-0.16, 0.36)** | **0.465** | **0.07**  **(-0.18, 0.32)** | **0.589** |
| Race x Sex | **Yes x Female** | **-0.37**  **(-1.28, 0.54)** | **0.429** | **-** | **-** |
| Ethnicity | **Mixed**  **Asian**  **Black African or Black Caribbean**  **Other ethnic group** | **-0.62**  **(-1.32, 0.07)**  **-1.64**  **(-2.52, -0.76)**  **-1.54**  **(-2.36, -0.72)**  **-0.84**  **(-2.01, 0.33)** | **0.079**  **<0.001**  **<0.001**  **0.161** | **-0.84**  **(-1.64, -0.04)**  **-1.78**  **(-2.62, -0.95)**  **-1.54**  **(-2.21, -0.86)**  **-1.11**  **(-2.31, 0.08)** | **0.039**  **<0.001**  **<0.001**  **0.069** |
| Race x Ethnicity | **Yes** **x Mixed**  **Yes x Asian**  **Yes x Black African or Black Caribbean**  **Yes x Other ethnic group** | **0.78**  **(-2.91, 1.35)**  **-0.65**  **(-1.88, 0.58)**  **-0.12**  **(-1.65, 1.41)**  **0 (omitted)** | **0.472**  **0.300**  **0.879**  **-** | **-** | **-** |
| Religion | **Christian**  **Muslim**  **Other religion** | **-0.09**  **(-0.34, 0.16)**  **-0.16**  **(-1.05, 0.72)**  **0.42**  **(-0.36, 1.19)** | **0.476**  **0.715**  **0.294** | **-0.08**  **(-0.34, 0.17)**  **-0.16**  **(-1.05, 0.73)**  **0.46**  **(-0.31, 1.24)** | **0.514**  **0.726**  **0.241** |
| Income | **£10,400 to £20,799**  **£20,800 to £36,399**  **£36,400 to £51,999**  **£52,000 or more** | **-0.11**  **(-0.57, 0.34)**  **-0.02**  **(-0.45, 0.48)**  **-0.05**  **(-0.55, 0.46)**  **-0.28**  **(-0.81, 0.26)** | **0.624**  **0.950**  **0.858**  **0.311** | **-0.12**  **(-0.58, 0.34)**  **0.01**  **(-0.47, 0.48)**  **-0.06**  **(-0.57, 0.45)**  **-0.29**  **(-0.82, 0.25)** | **0.598**  **0.981**  **0.814**  **0.288** |
| Parental education | **GCE A level**  **GCSE grades A-C**  **Qualification at level 1 and below**  **No qualification** | **-0.06**  **(-0.40, 0.28)**  **-0.56**  **(-0.91, -0.20)**  **-0.26**  **(-0.77, 0.24)**  **-0.27**  **(-0.74, 0.19)** | **0.741**  **0.002**  **0.306**  **0.251** | **-0.06**  **(-0.40, 0.28)**  **-0.55**  **(-0.90, -0.19)**  **-0.25**  **(-0.76, 0.25)**  **-0.26**  **(-0.73, 0.20)** | **0.714**  **0.002**  **0.326**  **0.265** |
| Smoking | **Used to smoke**  **Smokes occasionally (not every day)**  **Smokes everyday** | **0.20**  **(-0.14, 0.54)**  **0.18**  **(-0.21, 0.58)**  **0.60**  **(0.28, 0.93)** | **0.246**  **0.361**  **<0.001** | **0.20**  **(-0.14, 0.54)**  **0.17**  **(-0.22, 0.57)**  **0.60**  **(0.27, 0.92)** | **0.248**  **0.392**  **<0.001** |
| Alcohol use | **Monthly or less**  **2-4 times a month**  **2-3 times a week**  **4 or more times a week** | **-0.31**  **(-0.67, 0.04)**  **-0.72**  **(-1.11, -0.34)**  **-0.94**  **(-1.37, -0.51)**  **-0.41**  **(-1.03, 0.22)** | **0.085**  **<0.001**  **<0.001**  **0.202** | **-0.31**  **(-0.67, 0.05)**  **-0.72**  **(-1.11, -0.34)**  **-0.92**  **(-1.35, -0.49)**  **0.41**  **(-1.04, 0.21)** | **0.087**  **<0.001**  **<0.001**  **0.195** |
| Employment at age 25 | **No** | **1.11**  **(0.87, 1.35)** | **<0.001** | **1.10**  **(0.86, 1.34)** | **<0.001** |
| Education/work status at age 17 | **Doing apprenticeship or similar training**  **In a full-time paid job**  **Something else** | **-0.48**  **(-0.91, -0.05)**  **-0.46**  **(-1.07, 0.14)**  **0.29**  **(-0.06, 0.63)** | **0.030**  **0.136**  **0.102** | **0.48**  **(-0.91, -0.04)**  **-0.46**  **(-1.06, 0.15)**  **0.29**  **(-0.06, 0.63)** | **0.031**  **0.140**  **0.105** |
| BMI | **-** | **0.03**  **(0.01, 0.04)** | **0.004** | **0.026**  **(0.01, 0.04)** | **0.004** |
| GHQ-12 (pre-exposure) | **-** | **0.12**  **(0.08, 0.17)** | **<0.001** | **0.125**  **(0.08, 0.17)** | **<0.001** |

**Supplementary table 5: Crude and adjusted models of the association between racism and mental health using complete case analysis**

| Variable | Category | Model A* | | Model B** | | Model C*** | |
| --- | --- | --- | --- | --- | --- | --- | --- |
| GHQ-12 | | | | | | | |
|  |  | B | P-value | B | P-value | B | P-value |
| Racism | No  Yes | -  1.01  (0.52, 1.49) | -  <0.0001 | -  0.66  (0.18, 1.14) | -  0.007 | -  0.66  (0.17, 1.14) | -  0.008 |
| Overall life satisfaction | | | | | | | |
|  |  | OR | P-value | OR | P-value | OR | P-value |
| Racism | No  Yes | -  1.44  (1.10, 1.87) | -  0.007 | -  1.25  (0.95, 1.65) | -  0.108 | -  1.19  (0.96, 1.48) | -  0.103 |
| Self-Harm | | | | | | | |
|  |  | OR | P-value | OR | P-value | OR | P-value |
| Racism | No  Yes | -  1.31  (0.69, 2.51) | -  0.409 | -  1.06  (0.52, 2.15) | -  0.869 | -  1.11  (0.51, 2.41) | -  0.798 |
| Longstanding mental health illness | | | | | | | |
|  |  | OR | P-value | OR | P-value | OR | P-value |
| Racism | No  Yes | -  1.36  (0.85, 2.19) | -  0.204 | -  1.13  (0.68, 1.89) | -  0.639 | -  1.30  (0.79, 2.16) | -  0.305 |
